# Dietary Supplementation with Vitamin D, Fish Oil or Resveratrol Modulates the Gut Microbiome in Inflammatory Bowel Disease

**DOI:** 10.3390/ijms23010206

**Published:** 2021-12-24

**Authors:** Vivian Naa Amua Wellington, Vijaya Lakshmi Sundaram, Soudamani Singh, Uma Sundaram

**Affiliations:** Department of Clinical and Translational Sciences, Marshall University, Huntington, WV 25755, USA; wellington3@marshall.edu (V.N.A.W.); sundaram2@live.marshall.edu (V.L.S.); singhs@marshall.edu (S.S.)

**Keywords:** dietary supplements, vitamin D, fish oil, resveratrol, gut microbiome, IBD

## Abstract

Gastrointestinal health is influenced by the functional genes and metabolites generated by the human microbiome. As the volume of current biomedical and translational research indicates, the importance and impact of this ecosystem of microorganisms, especially those comprising the gut microbiome on human health, has become increasingly apparent. Changes to the gut microbiome are associated with inflammatory bowel disease (IBD), which is characterized by persistent intestinal inflammation. Furthermore, the lifetime dietary choices of their host may positively or negatively affect both the gut microbiome and its impact on IBD. As such, “anti-inflammatory” dietary supplements, their impact, and mechanisms in restoring gut microbiota homeostasis during IBD is an area of intensive research. Dietary supplementation may represent an important adjuvant treatment avenue for limiting intestinal inflammation in IBD. Overall, this review addresses the development of the gut microbiome, the significance of the gut microbiome in IBD, and the use of dietary supplements such as vitamin D, fish oil, and resveratrol in the mitigation of IBD-associated gut dysbiosis and intestinal inflammation.

## 1. Introduction

The human body is populated by a multitude of microorganisms known collectively as the microbiome. These microorganisms include bacteria, viruses, fungi, and their genetic materials, which all play a complex role within the human system. While earlier studies have suggested that there were significantly more bacterial cells than the number of human cells [1,2] in the body, a recent study has estimated the total number of bacteria to human cells to be almost equivalent, at 3.8 × 10^13^ to 3.0 × 10^13^ [3]. The human gastrointestinal tract possesses the highest concentration of microorganisms within the mammalian body [4]. Thus, the microorganisms that inhabit the gastrointestinal tract are known as the gut flora or gut microbiota.

The gut microbiota is critical in maintaining the proper functioning of mammalian intestinal physiology, including the regulation and control of the immune system, the elimination of toxic materials, the reinforcement of the intestinal barrier, and the catabolism of complex non-digestible carbohydrates [5,6]. These complex carbohydrates, found in dietary fiber, are converted into bioavailable metabolites to be absorbed as short chain fatty acids (SCFAs) in the intestine. Furthermore, the gut microbiota is a key contributor in regulating the lipid absorption and energy storage from one’s diet [5,6]. Therefore, changes in the abundance and make-up of the gut microbiota may lead to microbial imbalance or “dysbiosis”. In all, because the gut microbiota has a robust capability to affect human physiology, it is emerging as a new frontier in the modulation and possible treatment of inflammatory conditions such as inflammatory bowel disease (IBD).

While decades worth of research has examined the human gut microbiome, scientific studies have only recently begun to explore the complex and dynamic interplay between the gut microbiota, the immune system, diet, and disease. One of the first steps in understanding these interactions has been to characterize the “healthy human gut”. Numerous metagenomic studies, including the European Metagenomics of the Human Intestinal Tract (MetaHIT) and the Human Microbiome Project (HMP), have established the existence of stable patterns of microbial diversity and distribution in the healthy gut [7,8]. The aim of such studies is to compare gut microbiome patterns in the “healthy gut” to alterations in the gut signature seen in diseased conditions. 

In the healthy adult human gut, *Bacteroidetes* (this includes *Bacteroides* and *Prevotella*) and *Firmicutes* (including *Lactobacillus*, *Clostridium*, *Enterococcus*, and *Faecalibacterium*) are the most prevalent bacterial phyla [9]. At lower concentrations, bacteria of the genus *Actinobacteria*, *Proteobacteria*, and *Verrucomicrobia* are also significantly represented [9]. In contrast, the inflamed adult human gut possesses a different gut microbial signature, one often linked with intestinal inflammatory disorders such as IBD. Inflammatory bowel disease affects millions of individuals annually and is characterized by a reduction in bacterial diversity, an indicator of gut dysbiosis [10,11]. This reduction in bacterial diversity is not surprising considering the gut’s sophisticated immune system, developed partly through its constant exposure to microbial and ingested antigens from the diet [12,13]. For this reason, different dietary habits may affect gut microbial diversity, converging to have important consequences, such as IBD, on health. 

This article brings together three promising IBD remedies and highlights their proposed mechanisms of action, particularly through gut microbiota modulation. This review will examine the development and maturation of the gut microbiota, the contribution of the gut microbiota and its ecosystem to IBD, and current literature on the use of vitamin D, fish oil, and resveratrol as IBD treatments. Here, we critically explore and summarize how these supplements interact with the gut microbiome to exert their anti-inflammatory effects in cell, animal, and human models of IBD.

## 2. The Gut Microbiome: An Omnipresent System

The characterization of the human microbiome has not only enlightened, but also challenged the perceived significance of the microbiome’s role in health and disease. Current research has shown significant modification in the composition of the microbiota across the human lifespan.

### 2.1. The Early Development of the Microbiome

The sterile womb paradigm—the idea of the placenta ensuring fetal sterility in utero—has been contested since Tissier’s studies in the early 1900s. Currently, the idea is being abandoned [14]. This is due largely to extensive data demonstrating the presence of complex microbial populations in the meconium and feces of human preterm neonates [15,16], and in the umbilical cord blood of healthy neonates delivered via cesarean section [17]. Such observations suggest a role for the microbiome even before birth.

**Figure 1:**
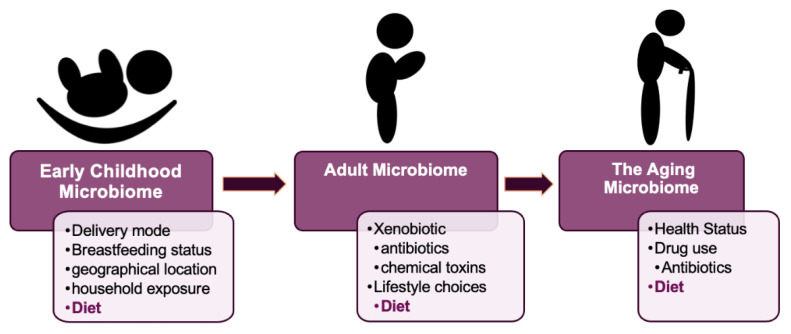
Factors affecting gut microbiota development through life.

On exposure to the postnatal environment, a host of microorganisms immediately colonize the human gastrointestinal tract. At this point, the infant’s microbiome is strongly influenced both by the mother’s diet and by microorganisms on the mothers’ skin, in breastmilk, and in the gastrointestinal tract [18,19] (Figure 1). A recent study focusing on the environmental determinants of diabetes in the young (TEDDY), demonstrated that the developing gut microbiome undergoes three distinct phases of progression: a developmental phase (months 3–14), a transitional phase (months 15–30), and a stable phase (months 31–46). Elevated levels of *Bifidobacterium* species (*B. breve* and *B. bifidum*) were associated with breastfeeding, while termination of breastfeeding resulted in advanced maturation of the microbiome. Other important covariates included birth mode as well as environmental factors including geographical location and household exposure [19].

The early colonization of the gut appears to be important not only for the child’s short-term development, but also for the child’s long-term health outcomes. Indeed, recent studies have established links between the infancy to childhood microbial population and the pathobiology of obesity, IBD, and type 1 diabetes [20,21,22,23]. Thus, the first few years of life provide a rare window of opportunity for influencing the gut microbiota in order to improve long-term health.

### 2.2. The Development of the Adult Microbiome

After about three years of age, the core gut microbiome composition is stabilized, and a gut microbiome signature appears to be established [24]. The gut microbiota composition at this point is similar to that of the adult gut microbiota. Although there is relative stability in the gut microbial population at this stage, the earlier mentioned TEDDY study [19] also revealed that populations of *Bacteroidetes* and *Actinobacteria* were less prone to fluctuations in microbial composition, whereas *Firmicutes* and *Proteobacteria* were substantially less stable. During this period of life, xenobiotics (external substances foreign to the body such as antibiotics and chemical toxins) and diet (Figure 1) are the two major factors which may modify the gut microbial composition over time, thus changing the distribution and relative abundance of particular bacterial groups [25]. Interestingly, accelerated transition into an adult-like gut microbiome upon the introduction of a solid diet had earlier been reported in a longitudinal, explorative study of a large cohort of Danish infants [26]. This 3-year study also reported an increase in the presence of butyrate-producing bacteria including *Clostridium* and *Roseburia*. This may be attributed to an increased need for their complex carbohydrate digestive capability. It must be noted that some studies have reported the presence of a distinct gut microbiota in adolescents, which is both different from the adult microbiota and heavily influenced by the adolescent’s lifestyle choices [27]. Such points exemplify the dynamic nature of the gut microbiota and underscore the need for further research.

### 2.3. The Gut Microbiome in Aging

Age-related processes can influence the gut microbiota. Consequently, the stability and complexity of a person’s gut microbiota decreases as the individual advances in age. This decrease usually occurs in tandem with the individual’s health, with an increased emergence of pathobionts [28]. Indeed, the gut microbiota composition of centenarians is not as diverse as that of adults as well as their elderly, but younger, counterparts [29]. In addition to other intrinsic (individual genetics and age-related physiological changes resulting from shifts in health status) as well as extrinsic factors such as drug use (antibiotic and otherwise), diet remains an influential contributor in determining the microbial population of the aging gut.

The deterioration of health may lead to a reduction or loss of motility and changes to the digestive capability of the individual. These changes may contribute to the consumption of an inadequate and often unbalanced diet [28]. As a result, age-related dietary changes may have a negative influence on the gut microbiota health of older persons and thence their longevity. Dietary therapies aimed at the gut microbiota of older persons could therefore be a good way to mitigate these negative consequences while also providing health advantages [30].

To summarize, microorganisms populating the human gastrointestinal tract influence human well-being pre-birth, post-birth (~0–3), in adulthood, and in old age. Therefore, the development of a “healthy” gut microbiome is crucial and may be affected both by environmental factors and lifestyle choices (Figure 1). Indeed, diet appears to be a major, constant, and unifying force for modulating the gut microbiome across human lifetime and will have a defining influence on intestinal diseases such as IBD. 

## 3. The Gut, Intestinal Inflammation, and Disease

Inflammation is a physiological reaction to an incursion of pathogenic and (or) nonpathogenic agents [31]. A healthy gut maintains a barrier that prevents the microbiota, undigested food particles, and toxins from leaking into the bloodstream. In the intestine, a diverse repertoire of cell types, including intestinal epithelial cells (IECs) and mucus-secreting goblet cells, serve not only to physically exclude commensal bacteria and strengthen this barrier, but also to integrate microbial signals [32,33].

The bulk of IECs are absorptive enterocytes, which are accompanied by specialized cell types such as hormone-secreting enteroendocrine cells and antimicrobial peptide-producing Paneth cells. These IECs interact constantly with surrounding immune cells and, like immune cells, can secrete chemical compounds such as cytokines and chemokines [34]. IECs may be compromised by a multitude of factors, including enhanced intestinal permeability due to an increasing presence of undesirable bacteria in the gut. These bacteria release enterotoxins, which trigger the development of immunosuppressive proteins, cause immune dysfunction, compromise intestinal epithelial cells, and disrupt energy metabolism, all of which contribute to intestinal inflammation [35]. Host toll-like receptors (TLRs), an important signaling mechanism, recognize a broad range of bacterial pathogen-associated molecular patterns and trigger acute inflammation by releasing inflammatory cytokines [36]. On the other hand, short-chain fatty acids and the Gram-negative bacteria-derived endotoxin lipopolysaccharides (LPS) are microbe-associated mediators of gut microbiota-inflammatory cross talk. These metabolites may drive both pro- and anti-inflammatory cascades [37]. Thus, the critical interaction between the gut microbiota and the host immune system is maintained by a finely curated intestinal architecture so as to ensure intestinal homeostasis.

The intestinal inflammatory cascade is initiated by cells that are already present in the intestinal epithelium, such as resident macrophages, dendritic cells, and mast cells. Signals denoting an incoming threat activate these cells. Activation is followed by the secretion of inflammatory mediators, which subsequently results in clinical signs of inflammation [38]. When the process of inflammation has been initiated, it proceeds until its source has been erased and the healing process can start. However, if the cause of inflammation cannot be eliminated, inflammation will continue, and may vary in intensity over time. In all, persistent inflammation due to immune system activation is undesirable and is part of the pathogenesis of IBD, along with changes in the gut microbial ecosystem [39].

### 3.1. Inflammatory Bowel Disease

Inflammatory bowel disease is a collective term that refers to a variety of disorders, primarily ulcerative colitis (UC) and Crohn’s disease (CD), which are characterized by chronic inflammation of the gastrointestinal tract. Unfortunately, a recently published observational study confirmed that IBD cases are rising in both older and younger demographics [40]. In children (2–17 years), there was a cumulative increase of 133%, from 33.0/100,000 in 2007 to 77.0/100,000 in 2016. For adults, (≥18 years), IBD prevalence increased by 123%, from 214.9 in 2007 to 478.4 in 2016 [40]. In CD, inflammation affects the entire digestive tract, but, for UC only, the colon is affected [41]. Interestingly, for children, CD is more prevalent than UC, while in adults, the prevalence of CD is similar to that of UC [40]. Typically, intestinal inflammation begins at puberty and remains throughout the affected person’s life [42].

#### 3.1.1. IBD and SCFA

**Figure 2:**
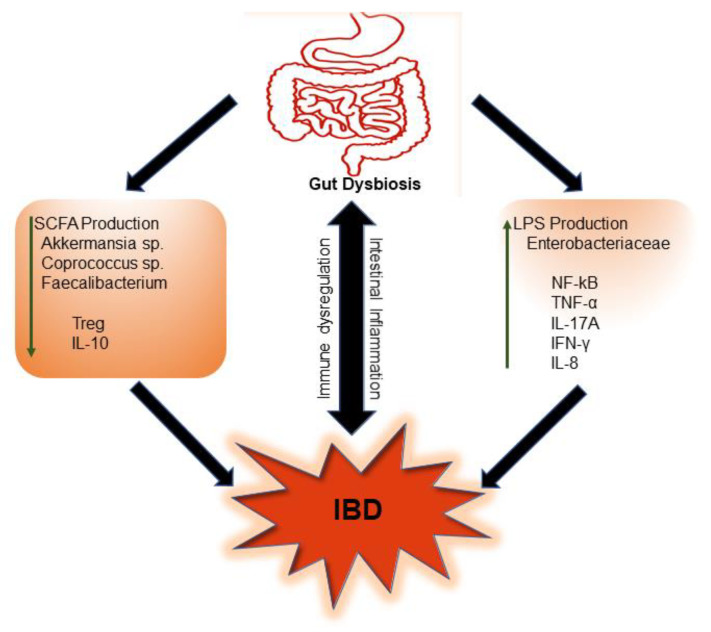
Gut microbiota dysbiosis, a common feature of inflammatory bowel disease (IBD). Dysbiosis of gut microbes leads to a reduction in short chain fatty acid (SCFA)-producing bacteria and an increase in lipopolysaccharide (LPS)-producing bacteria. Induction of consequent proinflammatory pathways and limited SCFA-induced anti-inflammatory response contribute to immune dysregulation, intestinal inflammation, and IBD. (IL, interleukin; Treg, T regulatory; NF-κB, nuclear factor kappa B; TNF, tumor necrosis factor; IFN, interferon).

Alteration of the gut microbiota has been shown to occur in subjects with newly diagnosed IBD. In fact, a prospective cohort study aimed to determine the association between gut microbiota dysbiosis and markers of disease activity [43]. By examining 19 treatments of naïve pediatric IBD subjects and 10 healthy controls, a study found that patients with CD, compared to the controls, had increased markers of inflammation and gut microbiota dysbiosis. Additionally, observable changes were reported in the bacteria of the genus, including *Akkermansia, Coprococcus, Fusobacterium, Veillonella, Faecalibacterium*, and *Adlercreutzia*. The significance of SCFA-producing bacteria such as *Faecalibacterium prausnitzii* during IBD is both critical and interesting. *Faecalibacterium prausnitzii* not only produces butyrate, but also converts acetate to butyrate, which is essential for colonic metabolism [44]. A functional consequence of the reduced *F. prausnitzii* in IBD is in the regulation of regulatory T cell (Treg) homeostasis [45], where butyrate stimulated Treg maturation. Butyrate also promoted proinflammatory T helper (Th)-17 to anti-inflammatory Treg cell balance so as to ameliorate intestinal inflammation [44]. Another study, which looked at *Prevotella intestinalis*, reported that *P. intestinalis*-associated gut microbiome dysbiosis worsened intestinal inflammation, which is linked to colitis in mice. This study also showed a significant reduction in the SCFA acetate and a consequent reduction in IL-18 production, a phenomenon which contributes to increased intestinal inflammation [32]. These findings reveal the significance of gut resident microorganisms and their metabolite (SCFA) in the induction and progression of IBD (Figure 2).

#### 3.1.2. IBD and Enterobacteriaceae

The pathogenesis of IBD appears to be related to *Enterobacteriaceae*, a broad genus of Gram-negative facultative bacteria which are elevated in the IBD [46]. For instance, CD has been linked to adherent-invasive *E. coli* (AIEC) [47], while UC has been linked to diffusely adherent *E. coli* (DAEC) [48]. Mirsepasi-Lauridsen et al. [46] also demonstrated that UC-associated *E. coli* p19A dissolved the tight junction protein occludin and disrupted the tight junctions in human intestinal colonic epithelial cell (Caco)-2 cells in vitro, followed by increased gut barrier permeability. Brubaker et al.’s [49] study using an experimental human challenge model showed that enterotoxigenic *E. coli* (ETEC) challenge resulted in significant intestinal inflammation both in participants with moderate to severe diarrhea and in asymptomatic participants. Furthermore, ETEC treatment resulted, post-administration, in elevated serum levels of IL-17A and interferon (IFN)-γ [49]. A useful explanation for why dysbiosis of these microorganisms may induce inflammation is presented in Kamada et al.’s paper [50]. The investigators suggested that pathogens within the family *Enterobacteriaceae*, such as *Citrobacter rodentium*, initially use virulence factors to induce intestinal inflammation, which subsequently confers a growth advantage for these pathogens in the intestinal lumen. The inflamed gut seen in IBD therefore provides an optimal environment for the overgrowth of bacteria of the genus *Enterobacteriaceae*.

#### 3.1.3. IBD and LPS

The contribution of LPS in the promotion of gut permeability and inflammation in relation to IBD has been widely reported [51,52]. Newly published data further suggests that LPS from different bacterial species may differentially trigger nuclear factor kappa B (NF-κB) and IL-10 as well as stimulate the production of proinflammatory IL-8 and tumor necrosis factor (TNF)-α [42]. Furthermore, the permeability of Caco-2 monolayers was differentially altered depending on the specific serotype of LPS used [42]. Interestingly, d’Hennezel et al.’s [53] recently published paper has suggested that LPS may actually have some immuno-inhibitory effects through silencing its cognate sensor, or toll-like receptor signaling [50]. These studies highlight the complexity of immunomodulatory microbiome–host interactions and underscore the need for further research in this field.

## 4. Diet, Gut Microbiota, and IBD

Diet, as we have seen in earlier sections of this review, is an important and constant theme in gut microbiome modification throughout the mammalian lifespan. Western diets, which are high in fat and sugar, have a negative impact on intestinal health due in part to the resulting gut microbiota dysbiosis. Consequently, a myriad of scientific research and discussion continues to explore avenues for remediating the gut microbiota dysbiosis associated with intestinal inflammation [54,55,56]. Broadly relevant dietary approaches in this field look at varying the ratio of proinflammatory to anti-inflammatory dietary products consumed. 

The benefits of dietary supplementation with prebiotics, probiotics and symbiotics in IBD has been extensively covered. The following sections examine current data for the use of other nutritional supplements which show broad promise for the treatment of intestinal inflammation in IBD. These include vitamin D, fish oil, and resveratrol.

### 4.1. Vitamin D

Vitamin D is a lipid-soluble vitamin which may be obtained by direct sun exposure, absorbed from vitamin D-rich foods, or from taking vitamin D supplements [57]. Vitamin D is metabolized in the liver to form 25(OH)D3, the major circulating metabolite, after which further renal hydroxylation generates 1,25(OH)2D3, the most potent metabolite [58].

#### 4.1.1. Vitamin D deficiency in IBD

Patients with IBD may be deficient in vitamin D. Additionally, medication commonly used by IBD patients (steroids, for example) may contribute to vitamin D deficiency [57,59]. Severe 25(OH)D3 deficiency is postulated to be a marker of a more aggressive clinical course of IBD [57,60]. One such study evaluated the influence of 25(OH)D3 status on the long-term clinical course of both CD and UC. This study, by Ham et al. [57], included 711 CD and 764 UC patients who had not had surgery prior to having their 25(OH)D levels assessed. Lowered 25(OH)D3 levels were linked with elevated disease activity scores and decreased c reactive protein (CRP) expression in both CD and UC patients. Additionally, Karima et al.’s [61] study showed that a daily dose of 2000 IU vitamin D improved serum 25(OH)D3 concentration as well as quality of life and decreased disease activity in UC patients with vitamin D deficiency. The investigators recommended that all patients with UC have their vitamin D levels checked because these patients may benefit from vitamin D therapy. A total of 50 patients with mild to moderate UC participated in this double-blind randomized clinical trial [61]. Hence, understanding the molecular mechanisms of these beneficial effects of vitamin D in IBD is important.

#### 4.1.2. Vitamin/VDR signaling in IBD

The active form of vitamin D—l,25(OH)2D3—functions predominantly through the vitamin D receptor (VDR) to activate downstream signaling cascades [62]. Indeed, the VDR is expressed by several cells in the innate and adaptive immune systems, and some of these cells produce 1,25(OH)2D3. Thus, disruption of the vitamin D/VDR signaling axis is detrimental to immune health [63].

**Figure 3:**
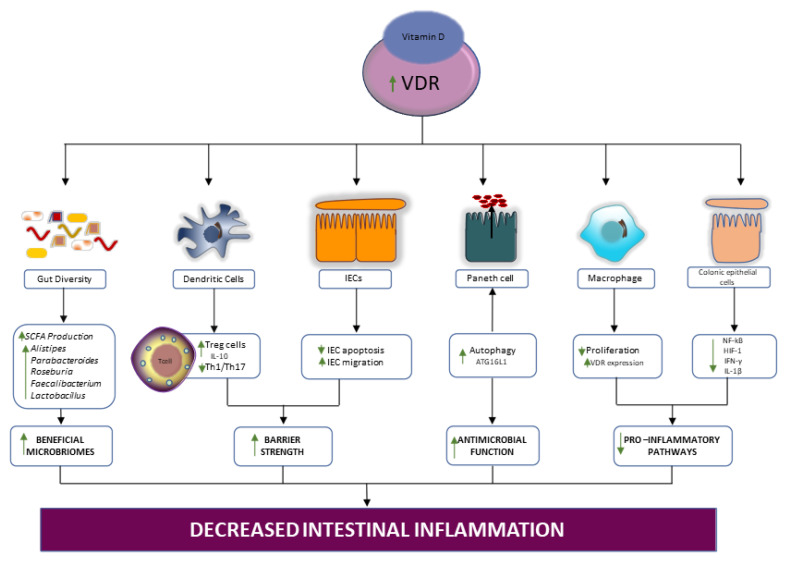
Action of vitamin D supplementation on the intestinal ecosystem. Vitamin D supplementation increases vitamin D/VDR signaling, which leads to an increase in SCFA-producing microbes, activation of proinflammatory Treg cells, inhibition of Th1/17 anti-inflammatory action, decreased intestinal epithelial cell (IEC) apoptosis and increased IEC migration, restoration of autophagy dependent Paneth cell action, increased macrophage proliferation and VDR expression, as well as reduction of anti-inflammatory cytokine action in colonic epithelial cells. (IEC, intestinal epithelial cell; IL, interleukin; Treg, T regulatory; Th, T helper; VDR, vitamin D receptor; NF-κB, nuclear factor kappa B; HIF-1, hypoxia inducible factor; IFN, interferon).

Vitamin D regulates dendritic function, promoting the development of immunomodulatory IL-10 producing Treg cells [64] (Figure 3). This immunomodulatory function appears to be related to VDR’s ability to inhibit inflammation-induced gut epithelial cell apoptosis. A study using an experimental colitis model with gut epithelial cell VDR knockout observed increased epithelial cell death, reduced intestinal barrier strength, and the consequent invasion of commensal gut microbes. Invasion of these microbes ultimately triggered robust mucosal Th1 and Th17 responses via the activation of dendritic cells [65]. Inhibition of inflammation-induced gut epithelial cell apoptosis by dendrite activation is significant, largely because earlier in vitro studies demonstrated that vitamin D/VDR signaling accelerates epithelial cell migration [66]. These reports suggest a key beneficial role for vitamin D/VDR signaling in the restoration of the damaged gut epithelial barrier seen in IBD (Figure 3). 

The vitamin D/VDR signaling axis is critical in autophagy. In fact, the deletion of intestinal epithelial VDR disrupts autophagy and its downstream cellular effects in colitis [67]. Wu et al. [67] demonstrated that VDR transcriptionally modulates ATG16L1, a regulator of autophagy and an IBD risk gene. Furthermore, low levels of VDR/vitamin D as well as ATG16L1 knockout resulted in abnormal Paneth cell function and gut microbial dysbiosis [67]. Thus, disruption of the anti-microbial activity of the Paneth cells may be a significant consequence of deficient vitamin D/VDR signaling in IBD.

The gut microbiota interacts with, and is modulated by, vitamin D and the VDR. Elevated VDR expression may limit microbial dysbiosis, promote SCFA production, minimize the secretion of proinflammatory cytokines, and improve intestinal barrier function [68]. By inhibiting NF-κΒ signaling and nuclear translocation, VDR reduces intestinal inflammation. Indeed, vitamin D-induced VDR–NF-κΒ interaction and disrupted LPS-induced macrophage proliferation [68]. Interestingly, another study showed that the modulation of the NF-κΒ pathway by vitamin D/VDR signaling suppressed hypoxia inducible factor 1 (HIF-1) overexpression in colonic epithelial cells. The reduction in HIF-1 inhibited IFN-γ and IL-1β overproduction in both in vitro and in vivo models of colitis [69]. 

#### 4.1.3. Supplementation with Vitamin D, the Gut Microbiota, and IBD

Supplementation with vitamin D directly impacts the gut microbiota. In a human study involving CD patients in remission, vitamin D supplementation resulted in significant shifts in gut microbiota composition, specifically in an increased presence of beneficial microbes, including *Alistipes, Parabacteroides, Roseburia*, and *Faecalibacterium* [70]. Earlier murine studies also demonstrated that the downregulation or loss of VDR resulted in a reduction in *Lactobacillus*, while *Clostridium, Bacteroides* and *Proteobacteria* populations were increased [71]. Therefore, vitamin D supplementation contributes to the restoration of gut microbiota homeostasis through the reestablishment of favorable gut bacteria.

While vitamin D supplementation is undoubtedly a promising therapeutic adjuvant in the recalibration of the gut microbiome and in the recovery from the resulting intestinal inflammation, studies such as Bashir et al.’s [72] found significant regional differences in the gut microbiota response to vitamin D supplementation. Vitamin D supplementation altered the gut microbiome in the upper GI tract, although no major gut microbiome alterations were observed in the terminal ileum, appendiceal orifice, ascending colon, and sigmoid colon of volunteers. The regional differences in the gut microbiome response to vitamin D underscores the need for ongoing research into the implication of these responses in IBD. 

Overall, the beneficial effects of vitamin D supplementation (Figure 3) are through a series of dynamic interactions involving the anti-inflammatory mediators produced by the cells of the innate and adaptive immune system, antimicrobial-producing Paneth cells, the regeneration of gut epithelium, and the restoration of beneficial gut resident microbiota.

### 4.2. Fish Oil

Fish oil contains omega-3 (n-3) long-chain polyunsaturated fatty acids (LC-PUFA). Although n-3 PUFAs can be obtained from various vegetables, the major source of n-3 is fish oil [73]. Omega-3 PUFAs are essential fatty acids which include docosahexaenoic acid (DHA), eicosapentaenoic acid (EPA), alpha-linolenic acid (ALA), as well as docosapentaenoic acid (DPA). Omega-3 PUFAs have significant influences on immune homeostasis and gut microbiota modulation, as discussed in the following sections [74].

#### 4.2.1. Omega-3-PUFA and the Gut Microbiota

**Figure 4:**
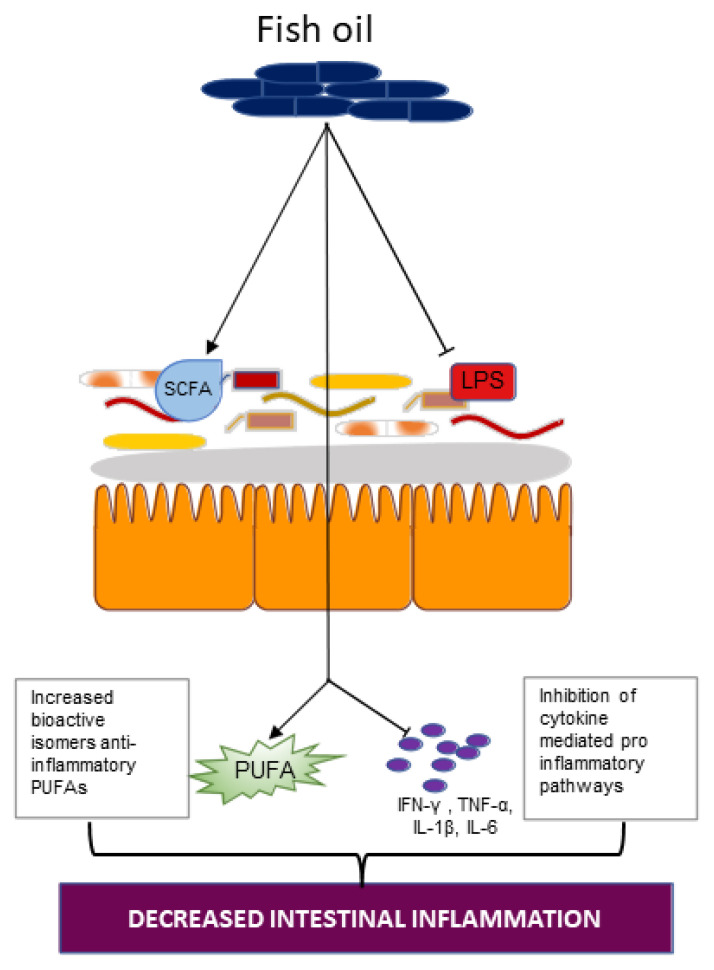
Action of fish oil supplementation on the intestinal ecosystem. Fish oil supplementation increases SCFA-producing microbes while decreasing LPS-producing gut bacteria. Fish oil supplementation may also encourage production of bioactive isomers of the anti-inflammatory polyunsaturated fatty acid (PUFA)-conjugated linoleic acid by the *Bifidobacterium* while inhibiting cytokine-mediated, proinflammatory pathways. These all lead to a reduction in intestinal inflammation. (SCFA, short chain fatty acid; LPS, lipopolysaccharide; PUFA, polyunsaturated fatty acid; IFN, interferon; TNF, tumor necrosis factor; IL, interleukin).

The effects of omega-3-PUFA on SCFA production in the gut microbiota is exemplified in a case study which examined the fecal sample of a 45-year-old man on a daily dose of 600 mg omega-3 PUFA for 14 days [75]. After two weeks on the omega-3-rich diet, investigators observed significantly increased bacteria of the genera *Eubacterium, Roseburia, Anaerostipes, Coprococcus, Subdoligranulum,* and *Pseudobutyrivibrio,* which are all directly linked with SCFA production (Figure 4) [75]. In a different study, investigators fed either a fish oil or a lard diet to mice. Mice fed fish oil had higher levels of *Lactobacillus, Bifidobacterium,* and *Akkermansia muciniphila* compared to mice on the lard diet [76]. Hence, the administration of omega-3-PUFAs affects the composition of the gut microbiota and ameliorates dysbiosis of the microbiota *Firmicutes*/*Bacteroidetes* ratio, which is associated with decreased SCFA generation and increased gut permeability.

The level of omega-3 PUFAs in the blood is linked to the composition of the gut microbiota [77]. Higher concentrations of the omega-3 fatty acids EPA and DHA were found after a daily dose *Bifidobacterium breve* was administered to mice and pigs and resulted in an altered profile of PUFAs. Note that *Bifidobacteria* had previously been shown to produce bioactive isomers of an anti-inflammatory PUFA, namely conjugated linoleic acid (Figure 4) [78]. Furthermore, the oral administration of commensal *Bifidobacteria* reduced levels of the proinflammatory cytokines TNF-α and IFN-γ [78]. These studies suggest that reciprocal interaction between the gut microbiota, particularly through *Bifidobacteria*, and omega-3, are important in halting a proinflammatory response. Another study reported that both DHA-phospholipid and EPA-phospholipid successfully countered intestinal inflammatory dysfunction via a reduction in the intestinal levels of IFN-γ, TNF-α, IL-1β, and IL-6, brought on by chronic stress exposure in male BALB/c mice. As such, omega-3 administration may limit proinflammatory action by inducing the production of anti-inflammatory PUFAs by the gut microbiota and could serve as a functional food material [79]. Omega-3 PUFAs also impeded the generation of proinflammatory cytokines through suppressing LPS-induced proinflammatory action. By regulating the inhibitor of κB (IκB) phosphorylation, ALA, EPA, and DHA mediate their anti-inflammatory effects, thus inhibiting all NF-κB pathways induced by LPS [80].

The gut microbiota is therefore influenced by omega-3 PUFAs in a variety of ways, including modulating the diversity of gut microbes, the consequent elevation of the SCFA level, inducing the production of anti-inflammatory PUFAs, and regulating the levels of proinflammatory mediators including LPS (Figure 4) [81].

#### 4.2.2. Omega-3 Supplementation in IBD

So far, studies have shown that omega-3 PUFA supplementation has several beneficial effects, including protection against IBD [82]. A large European study involving 229,702 participants found an inverse association between increased consumption of dietary DHA and the development of Crohn’s disease [83]. Moreover, Meister et al. [84] treated tissues collected from CD and UC patients with fish oil-enriched diets. Fish oil treatment resulted in a significantly increased ratio of IL-1ra to IL-1β in UC [84]. Interleukin-1ra is the receptor antagonist of IL-1 and can competitively bind with IL-1 receptor, thereby blocking cell activation by the proinflammatory IL-1. Furthermore, *Bifidobacteria, Lactobacilli, Prevotella spp*., and *Roseburia spp*. were significantly increased with fish oil supplementation, while *Enterobacteriaceae* and *Enterococcus spp*., both related to gut inflammation, were markedly decreased. Meister et al.’s results are consistent with previously mentioned studies which suggest a repression of pro-inflammatory pathways on fish oil/omega -3 administration. In addition, a meta-analysis and systemic review examining the association between fish consumption and the dietary intake of n-3 PUFAs with the risk of IBD was recently published [85]. Findings from the meta-analysis revealed an inverse relationship between fish/omega-3 consumption and the risk of CD as well as UC. However, another recently published meta-analysis reported the negligible effect of ALA in alleviating IBD risk, while supplementation with EPA and DHA appeared to increase the risk of IBD diagnosis [86]. 

In summary, dietary fish oil-derived omega-3 PUFAs interfaces with the gut microbiota to promote immune system homeostasis. Therefore, dietary supplementation with fish oil may improve disease outcomes for individuals with IBD. Nonetheless, there is still the need to better understand the underlying dynamics governing the relationship between supplementation with fish oil, the gut microbiota, and the alleviation of IBD-associated intestinal inflammation.

### 4.3. Resveratrol

Polyphenols such as quercetin, curcumin, and resveratrol are among important phytochemicals that have been shown to have anti-inflammatory health benefits [87]. Resveratrol is a dietary polyphenol, abundant in grapes and red wine, that has both anti-oxidative and anti-inflammatory properties [88]. Around 90% of ingested resveratrol enters the colon in its native form and then undergoes gut fermentation [89]. Therefore, what develops is a bidirectional interaction between resveratrol and the gut microbiota: there is resveratrol biotransformation by the gut microbiota and reciprocal targeting of the gut microbiota by resveratrol, so as to maintain gut homeostasis [90]. Resveratrol’s reciprocal relationship with the gut microbiota may account for the beneficial effects of resveratrol administration on in vitro and in vivo models of IBD. 

#### 4.3.1. Resveratrol and Gut Microbiota

**Figure 5:**
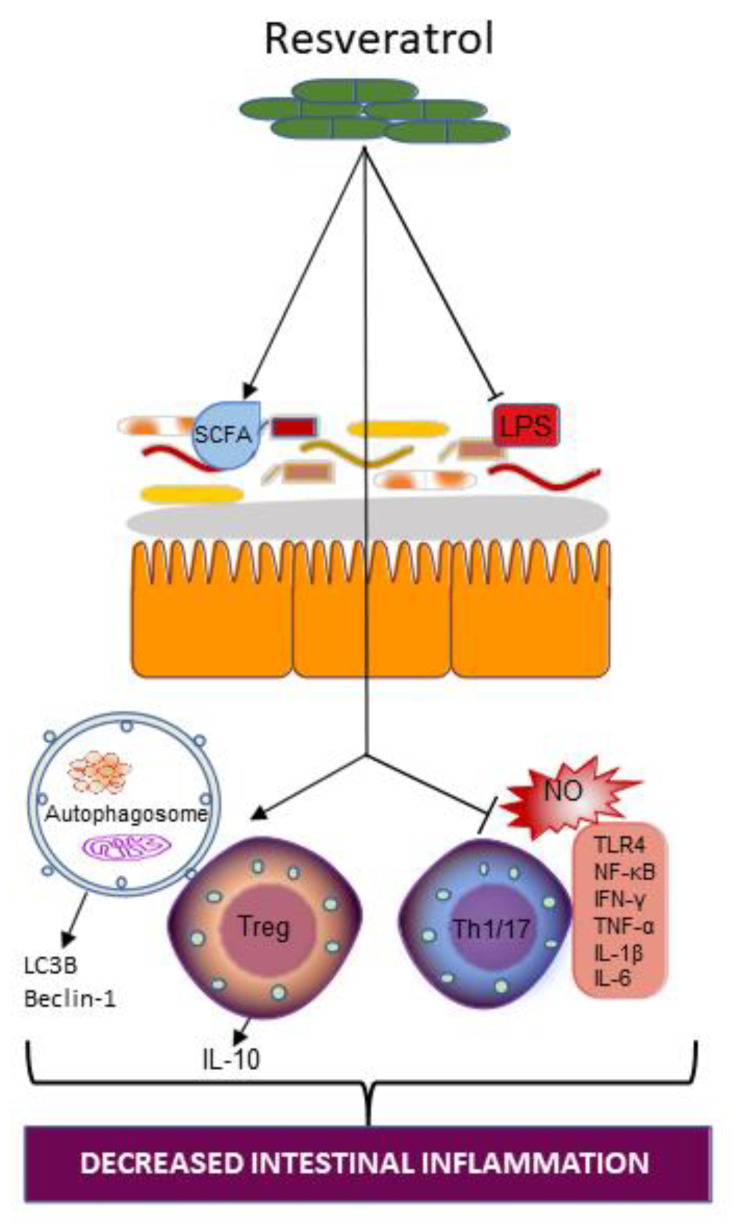
Action of resveratrol supplementation on the intestinal ecosystem. Resveratrol supplementation increases SCFA-producing microbes while decreasing LPS-producing gut bacteria. Resveratrol supplementation may also restore autophagy by increasing the number of autophagosomes and inducing the expression of microtubule-associated protein 1A/1B-light chain 3 (LC3) and Beclin-1, both important proteins in autophagy. Resveratrol supplementation may also interrupt Th1/17 and cytokine-dependent proinflammatory pathways, nitric oxide dependent pro-oxidative pathways, as well as disrupt toll-like receptor (TLR) 4 signaling. (SCFA, short chain fatty acid; LPS, lipopolysaccharide; LC, light chain; IL, interleukin; Treg, T regulatory; Th, T helper; NO, nitric oxide; NF-κB, nuclear factor kappa B; IFN, interferon; TNF, tumor necrosis factor).

Resveratrol administration has an effect on gut microbiota diversity. *Enterococcus faecalis* growth was inhibited by resveratrol therapy, but *Bifidobacterium* and *Lactobacillus* growth was increased [91]. An earlier study of the effect of dietary administration of resveratrol (1 mg/kg/day) in a dextran sulphate sodium (DSS)-model of colitis showed the substantially reduced deterioration of colon wall structure with resveratrol treatment [92]. Also reported in that same study was a decrease in inducible nitric oxide (NO) synthase expression and increased abundance of *Bifidobacterium* and *Lactobacillus*, which may account for the restoration of the colonic barrier. In Yao et al.’s [93] study using a UC model of IBD, investigators reported that resveratrol-mediated colitis relief was due to the reversal of microbial dysbiosis caused by colitis and that the microbiota protects the host from colonic inflammation by inducing Treg cells and suppressing inflammatory Th1/Th17 cells (Figure 5). Likewise, resveratrol treatment decreased the levels of the intestinal mucosal proinflammatory cytokines IL-6 and IL-17 and stimulated levels of the anti-inflammatory cytokine IL-10 [94]. Furthermore, 2,4,6-trinitrobenzenesulfonic acid (TNBS)-induced reduction in *Akkermansia mucinphilia* and butyric acid was also alleviated upon treatment of mice with resveratrol [94]. These studies illustrate that the significant interaction between resveratrol and the gut microbiota may be necessary in promoting a proinflammatory to anti-inflammatory balance within the gut.

#### 4.3.2. Resveratrol Supplementation on Intestinal Inflammation in IBD

In a study investigating the impact of resveratrol on LPS-induced inflammatory response, the authors found that resveratrol treatment of human intestinal Caco-2 and SW480 (a colon adenocarcinoma cell line) cell lines resulted in a reduced expression of iNOS, suppressed NO production, and decreased TLR4 expression [88] (Figure 5). Further investigation into the possible mechanisms involved in this process revealed that resveratrol, by the phosphorylation and degradation of the IκB complex in LPS-stimulated intestinal cells, interferes with the activation of NF-κB-dependent molecular mechanisms [88]. Accordingly, the benefits of resveratrol for IBD may involve the disruption of LPS-induced pro-oxidative and inflammatory pathways.

Results from a randomized double-blind clinical trial involving 50 individuals with mild to moderate UC, who were supplemented with either a 500-mg resveratrol or a placebo for six weeks, revealed improvement in salient inflammatory markers [95]. Resveratrol supplementation resulted in a significant reduction in plasma levels of TNF-α, high sensitivity-CRP, and NF-κB activity (all markers of a proinflammatory state) compared to the placebo [91]. Recently published data also suggested that the beneficial effects of resveratrol treatment in the amelioration of intestinal inflammation in IBD may be through an autophagy-dependent pathway [96]. Indeed, resveratrol increased the expression of microtubule-associated protein 1A/1B-light chain 3B (LC3B) and Beclin-1, both important components of the autophagy pathway, as well as the number of autophagosomes (Figure 5), while promoting intestinal barrier repair. Furthermore, resveratrol may act in a dose-dependent manner. For example, the previously discussed Yao et al. study [90] also indicated that the effect of resveratrol on Treg/Th17 modulation was dose-dependent. A low dose of 50 mg/kg resveratrol regulated Treg/Th17 balance mainly by the reduction in the number of Th17 cells, whereas high doses of resveratrol (100 mg/kg) regulated Treg/Th17 balance by the downregulation of the number of Th17 cells as well as an upregulation of the number of Treg cells. Thus, resveratrol supplementation presents a dynamic modulator of gut inflammation in IBD.

The studies highlighted here (as summarized in Table 1) provide some understanding of the functional consequences of resveratrol–gut microbiota interaction in the alleviation of a proinflammatory state: promoting beneficial microbe growth, anti-inflammatory processes, and inhibiting proinflammatory pathways (Figure 5).

## 5. Conclusions and Future Directions

Dietary habits are important in the development of a host-specific gut microbial community in humans. Therefore, diet and dietary adjuvants which encourage the development of an optimal gut microbial community will remain a promising area of research into the alleviation of IBD-related gut inflammation.

Future IBD-related studies should critically explore and expand on the potential for combination therapies which include vitamin D, fish oil, and resveratrol. This may serve in exploiting the different modes of action of these dietary adjuvants in the mitigation of IBD-associated inflammation and microbial dysbiosis. Futhuremore, better designed basic and clinical studies which explore important questions such as “what is the ideal therapeutic dose of these remedies” are needed.

Collectively, the studies presented in this review broadly support the use of vitamin D, fish oil, and resveratrol in the treatment of gut microbiota-mediated inflammation in IBD. However, there is a need for a better understanding of the underlying processes governing the relationship between dietary supplementation with vitamin D, fish oil, or resveratrol, and the gut microbiota. Recognition of how this interplay aids in the alleviation of IBD-associated intestinal inflammation will help in formulating optimal therapeutic interventions of the future.

## Figures and Tables

**Table 1:** Summary of Key Vitamin D, Fish Oil, and Resveratrol Studies.

Study	Study Type	Participants	Dose	Key Conclusions/Recommendations
	**Vitamin D**			
[57]	Clinical Retrospective study	711 Crohn’s disease (CD) 764 ulcerative colitis (UC) patients	-	Severe 25(OH)D deficiency may be a marker of a more aggressive clinical course of inflammatory bowel disease (IBD)
[60]	Retrospective Observational study	155 Crohn’s disease 77 ulcerative colitis patients	-	25(OH)D supplementation in deficient IBD patients is recommended
[61]	Double blind randomized clinical trial	50 patients with mild to moderate UC	1000 or 2000 IU/day vitamin D for 12 weeks.	Recommend assessment of the vitamin D status in all patients with UC because they may benefit from vitamin D therapy.
[70]	Prospective, longitudinal, controlled interventional analysis	7 CD patients with vitamin D deficiency, 7 healthy control (HC) patients	20 000 IU daily (day 1–3, then every other day) for 4 weeks	Vitamin D has a specific influence on the bacterial communities in CD, but not in HC.
	**Fish oil**			
[83]	Prospective study	229,702 participants recruited between 1991 and 1998.	-	Higher quintiles of docosahexaenoic acid (DHA) intake were inversely associated with development of CD
[84]	In vitro study	Biopcies of 4 patients with active CD 7 active UC patients 4 control patients	fish oil supplemented enteral elemental diet (diluted 1:20, 1:10, and 1:5) for 24 h	Dietary treatment of UC may be possible
[85]	Meta-analysis of observational studies	participants (2002 cases of IBD)	-	Negative association between fish consumption and the risk of CD. Inverse association between dietary\n-3 polyunsaturated fatty acids (PUFAs) and risk of UC
[86]	Meta-analysis of randomized controlled trials	41,751 participants	-	Supplementation with PUFAs has little or no effect on prevention or treatment of IBD.
	**Resveratrol**			
[92]	In vivo study	dextran sulphate sodium (DSS)-induced colitis rats	1 mg of resveratrol/kg/day for 25 days	Resveratrol as a beneficial dietary compound in intestinal inflammation is possible
[93]	In vivo study	DSS-induced colitis mice	50 mg/kg per day group and resveratrol 100 mg/kg per day group for 7 days	The therapeutic efficacy of resveratrol in UC is dose dependent.
[94]	In vivo study	2,4,6-trinitrobenzenesulfonic acid (TNBS)-induced colitis mice	100 mg/kg/day for 5 days	Resveratrol-mediated attenuation of colitis results from reversal of microbial dysbiosis induced during colitis.
[88]	In vitro study	Lipopolysaccharide-treated intestinal cells	30, 40, 50 μM resveratrol for 1 h	Resveratrol can reduce LPS-induced inflammatory responses in intestinal cells.
[95]	Randomized, double-blind, placebo-controlled study	50 patients with active mild to moderate UC	500-mg resveratrol or placebo capsule for 6 weeks.	Supplementation with 500 mg resveratrol can improve quality of life and disease clinical colitis activity.
[96]	In vivo study	DSS-induced chronic colitis mice	resveratrol 100 mg/kg per day by gavage	Resveratrol may alleviate intestinal mucosal barrier dysfunction in DSS-induced UC mice by enhancing autophagy
